# The Effect of Heat Treatment on Cow’s Milk Protein Profiles

**DOI:** 10.3390/foods11071023

**Published:** 2022-03-31

**Authors:** Jozef Čurlej, Peter Zajác, Jozef Čapla, Jozef Golian, Lucia Benešová, Adam Partika, Alexander Fehér, Silvia Jakabová

**Affiliations:** 1 Institute of Food Sciences, Faculty of Biotechnology and Food Sciences, Slovak University of Agriculture in Nitra, Tr. Andreja Hlinku 2, 949 76 Nitra, Slovakia; jozef.curlej@uniag.sk (J.Č.); jozef.capla@uniag.sk (J.Č.); jozef.golian@uniag.sk (J.G.); xbenesova@uniag.sk (L.B.); xpartika@uniag.sk (A.P.); silvia.jakabova@uniag.sk (S.J.); 2 Institute of Environmental Management, Faculty of European Studies and Regional Development, Slovak University of Agriculture in Nitra, Tr. Andreja Hlinku 2, 949 76 Nitra, Slovakia; alexander.feher@uniag.sk

**Keywords:** milk, heat treatment, denaturation, whey protein, casein, protein fraction

## Abstract

Milk is a food of high nutritional value processed by heat treatment. Heat treatment of milk is a technological process designed to inhibit the growth of microorganisms and extend the shelf life of products. The heating process directly affects the molecular structure of whey proteins by the process of denaturation. It leads to the formation of a whey protein–casein polymer complex. Based on these facts, milk heat-treatment conditions should be controlled during milk processing. This work focuses on describing the whey protein denaturation process and formation of the complex of whey protein with casein. The effect of heat treatment on individual milk protein fractions alpha-casein (α-cas), beta-casein (β-cas), kappa-casein (κ-cas), beta-lactoglobulin (β-lg) and alpha-lactalbumin (α-la) was studied by SDS-PAGE. Formation of the whey protein–casein polymer complex increased significantly (*p* < 0.05) on increasing the temperature and duration of the heat treatment.

## 1. Introduction

Milk is an important source of nutrients for humans, including milk proteins [1]. Traditionally, milk proteins are classified into two major categories. The first and most abundant is the casein family, which consists of several fractions (αs1, αs2, β and ĸ). Most of them exist in a colloidal particle known as the casein micelle. The second protein group in milk is whey proteins, including heat-sensitive, globular, water-soluble proteins and enzymes [2,3,4]. The protein content of milk can be influenced by the proteolytic activity of enzymes present in the milk [5], the breed, lactation period and feed [6,7]. Moreover, individual protein fractions can be affected by the heat treatment of milk [8,9], which is specifically intended to lower the microbial risk [10,11]. Low-temperature, long-time pasteurization (LTLT, 63 °C/30 min) or high-temperature, short-time pasteurization (HTST, 72 °C/15 s or 85 °C/2–5 s) are generally used in the dairy industry. Ultra-high temperature processing (UHT, 135–150 °C for seconds) combined with aseptic packaging can be applied to prolong the milk’s shelf life. Generally, heat treatment, as the most common processing method in the dairy industry, is a critical process to eliminate the pathogens and other microorganisms in raw milk; it aims to ensure food safety and extend the shelf life of milk products [12,13] and to fulfil legislative requirements [14]. Heat treatment may change the structure of milk proteins [8], depending on pH and temperature. Whey proteins can interact with themselves or caseins [15,16,17,18,19,20].

Denaturation involves the unfolding of whey proteins to expose reactive functional groups, such as the free thiol groups in β-lactoglobulin. These functional groups can react with other denatured whey proteins, caseins or ĸ-casein [19,20].

Although a polymerization reaction cannot occur with α-lactoglobulin due to the absence of a free thiol group, the disulphide bonds inside will also lead it to denature due to a thiol–disulphide bond exchange reaction [21]. Available proteins determine the composition, structure and reactivity of aggregates formed in heat-treated milk systems. The casein to whey protein ratio is significantly impacted by the particle size, viscosity and heat stability observed in dairy products [22]. Changing this ratio can alter the kinetics of protein denaturation reactions [23]. The addition of casein (β and ĸ) can improve heat stability through a protective chaperone-like effect, preventing irreversible whey protein denaturation and aggregation during thermal processing [24,25]. Casein–whey protein aggregates exhibit better heat stability than aggregates consisting of whey proteins alone [26,27]. In the production of dairy products, it is possible to preheat milk to bind part of the whey proteins to caseins. This operation reduces whey protein losses, increases the thermal stability of the milk, and changes the physical characteristics of dairy products [17,28].

Milk protein denaturation is well described in several articles [2,4,8,9]. Therefore, this article confirms the already existing facts present in these studies. However, in this article we have focused on the quantification of denaturation of individual protein fractions, especially the beta casein. We are using this protein fraction and the cleavage products (γ2 and γ3-caseins markers) of this fraction for the detection of the adulteration of traditional Slovak bryndza cheese (mixture of ewe’s lump cheese by cow’s lump cheese) [4]. This traditional cheese has protected geographical indications PGI-registered in the European Union [29]. We quantified the denaturation of beta casein in order to include the influence of this pasteurization in the calculation of the uncertainty of the measurement of the isoelectric focusing of the casein method [4].

Our study’s main goal was to determine the effect of different heat-treatment conditions on the concentration of individual protein fractions. Our study examined the rate of milk protein denaturation under different heat-treatment conditions. The relationship between the degree of milk protein denaturation and heating conditions was established through polynomial regression equations.

## 2. Materials and Methods

### 2.1. Design of the Experiment

The experiment was performed following this scheme:

- Analysis of the protein content in raw milk (FTIR spectroscopy);
- Heat treatment of milk (Experiment 1—heating time: 10 min, 20 min and 30 min, heating temperature: 65 °C, 75 °C, 85 °C, 95 °C, 100 °C; Experiment 2—heating time: 5, 10, 15, 20, 25, 30 min, heating temperature: 75 °C, 85 °C, 95 °C);
- Analysis of the protein content in heat-treated milk (FTIR spectroscopy);
- Skimming of heat-treated milk samples;
- Analysis of the protein content of the supernatant of skimmed heat-treated milk samples (UV spectroscopy);
- Analysis of whey protein and casein complex denaturation (SDS-PAGE);
- Densitometric analysis of stained gels;
- Determination of denaturation degree of individual proteins fractions (%);
- Statistical analysis.

### 2.2. Collection of Milk Samples

Fresh raw cow’s milk was sampled from the selected farm of PD Vráble, Slovakia, and transported to our laboratory following international standards [30] at 0–5 °C to inhibit microbial and enzymatic activity.

### 2.3. Heat Treatment of Milk Samples

Aliquots (500 mL) of raw milk were heated and slightly mixed in a water bath at temperatures of 65, 75, 85, 95 and 100 °C. The heating time was set to 10, 20 and 30 min. Other aliquots (500 mL) of raw cow’s milk were heated at 75, 85 and 95 °C, respectively. The heating time was set to 5, 10, 15, 20, 25 and 30 min. The milk was subsequently cooled down to ambient temperature, and the protein content in heat-treated milk samples was analyzed on the same day by FTIR spectrometry (Bentley Instruments, Chaska, MN, USA).

### 2.4. Skimming the Heat-Treated Milk Samples

In detail, the samples were put into a 1 mL centrifuge tube and skimmed at 10,000 rpm at 4 °C for 25 min using a centrifuge (Eppendorf 5702 R, Avantor, Bratislava, Slovakia).

### 2.5. Analysis of Protein Content

The total protein content (TPc) of raw and heat-treated milk subjected to different temperatures and times was investigated using a DairySpec FT analyser (Bentley Instruments, Chaska, MN, USA). The instrument was calibrated using a set of calibration samples (Actalia-Cecalait, Poligny, France) with a range of fat (1.9% to 5.4%), protein (2.1% to 4.1%), lactose (4.5% to 5%, 8%) and dry matter (9.7% to 14.6%).

UV spectrophotometry was used to analyze the TPc in skimmed heat-treated milk samples [9,31]. The supernatant was diluted ten times with distilled water; the absorbance of the supernatant at 260 nm and 280 nm was measured using an Epoch UV spectrophotometer (Agilent, Santa Clara, CA, USA). TPc was calculated by the following Formula (1):

TPc (mg/mL) = 1.45 × A_280_ − 0.74 × A_260_ (1)

### 2.6. SDS-PAGE Analysis of Protein Fraction Denaturation

The change in protein fractions and formation of the whey protein and casein complex was analyzed by horizontal SDS-PAGE.

Firstly, each supernatant subsample was dissolved with the reducing sample buffer. Secondly, the dissolved subsamples were heated at 95 °C for 5 min to reduce the disulphide bonds. Subsequently, the samples were cooled to room temperature and applied to the gel. The volume of each subsample applied to the gel was 15 µL (the concentration of proteins in the applied subsample was diluted to 8 µg/mL).

Other chemicals used for the stock buffer and the sample buffer were of PA grade (Serva, Heidelberg, Germany).

Sample stock buffer (50 mL): 3.0 g Tris, distilled water. The pH should be set to 7.5 by adding approx. 1.4 mL acetic acid. Storage: 3 months at +2 °C to +8 °C [32].

Sample buffer—reducing (50 mL): 5.0 mL sample stock buffer, 0.5 g SDS, 77 mg DTT, 5 mg Bromophenol Blue, distilled water. Use fresh [32].

We used a precast Excellent Gel Kit NF 15% for 1D SDS-PAGE (250 × 125 × 0.45 mm, 25 slots for 15 µL; Serva, Heidelberg, Germany) and followed the Serva protocol [33]. The kit components included: four pieces of gel, 250 mL of SDS Anode Buffer (blue), 250 mL of SDS Cathode Buffer (white) and electrode wicks.

For horizontal electrophoresis, we used BlueHorizon HPE™ and Blue Power TM 400 Prime Power Supply (Serva, Heidelberg, Germany).

The running conditions were [32]: 15 °C, total running time 2 h 30 min. Phase 1 (150 V, 30 mA, 10 W, 45 min), Phase 2 (700 V, 42 mA, 30 W, 45 min), Phase 3 (1000 V, 50 mA, 60 W, 1 h).

A protein molecular weight marker range 6.5–200 kDa (200, 116, 97.2, 66.4, 44.3, 29, 20.1, 14.3 and 6.5 kDa) was purchased from Takara Bio (Shiga, Japan).

The gel was stained with a 0.23% solution of Coomassie^®^ Brilliant Blue R 250 (Serva, Heidelberg, Germany) for 90 min and subsequently destained in a 5% methanol (Centralchem, Bratislava, Slovakia): 7% acetic acid (Centralchem, Bratislava, Slovakia) solution. Treated gels were captured using an Azure 200 blue light white light UV platform (Serva, Heidelberg, Germany).

### 2.7. Densitometric Analysis

Firstly, the protein bands of alpha-casein (α-cas), beta-casein (β-cas), kappa-casein (κ-cas), beta-lactoglobulin (β-lg) and alpha-lactalbumin (α-la) were identified on the gel based on the molecular weight [9]. Densitometric analysis of these bands was then performed using the gel imaging software AzureSpot version 2.2.167 (Serva, Heidelberg, Germany). 

The amount of protein in the diluted supernatant subsamples applied to the gel was calculated by recalculating the amount of protein (determined by UV spectrophotometry) with the applied dilution. Consequently, the used protein content was assigned to the total density of all bands in the line. The concentration of individual protein fractions (g/100 mL) was automatically calculated using the software based on the density of the individual bands.

The results of the densitometric analysis were plotted on graphs, and the polynomial functions for denaturation of individual protein fractions were calculated in Microsoft Excel (Office 365, v. 2021).

Denaturation degree represents the percentage (%) of increase or decrease in the individual protein fraction depending on the heating temperature and time, and can be calculated by the following the Formula (2):

(2) $$\mathrm{Denaturation}\;\mathrm{degree}\;(\%)=\frac{\mathrm{individual}\;\mathrm{protein}\;\mathrm{fraction}\;\mathrm{content}\;\mathrm{in}\;\mathrm{heat}\;\mathrm{treated}\;\mathrm{milk}\;(g/100\;\mathrm{mL})}{\mathrm{individual}\;\mathrm{protein}\;\mathrm{fraction}\;\mathrm{content}\;\mathrm{in}\;\mathrm{raw}\;\mathrm{milk}\;(g/100\;\mathrm{mL})}\;\times \;100\%$$

### 2.8. Statistical Analysis

The statistical analysis was performed using XLSTAT, version 1 April 2021. (Addinsoft, Paris, France). Obtained data were reported as mean values in g/100 mL ± SD. Experiments were performed in triplicate. Firstly, the Shapiro–Wilk normality test tested the normality of data for both experiments. As the computed *p*-values for each protein fraction were greater than the significance level of *p* = 0.05, one cannot reject the null Hypothesis H0 for both experiments. The data had a normal distribution. Secondly, the effect of heating temperature during the defined time on the content of protein fractions was analyzed. The significance of the differences between the mean values for individual protein fractions at different heating temperatures during the defined time was analyzed by one-way ANOVA and Tukey’s test (*p* < 0.05). Results (Table 1) assigned with a different letter are statistically significantly different. The significance of the differences between the mean values for individual protein fractions at different heating temperatures and different heating times was assessed by two-way ANOVA (*p* < 0.05). Thirdly, the same statistical procedure was used to analyze the effect of heating temperature and time on the content of the protein fractions (Table 2). The results of two-way ANOVA for both experiments are presented in Table 3.

## 3. Results

**Figure 1:**
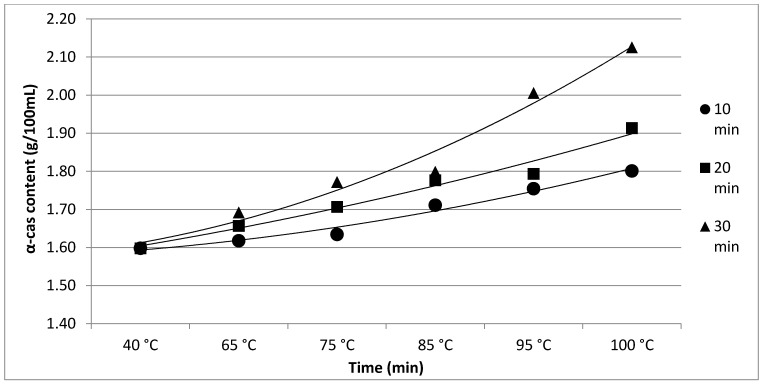
Effect of heating time and temperature on α-cas content. Equations: 10 min (y = 0.0043x^2^ + 0.0127x + 1.5763; R^2^ = 0.9794), 20 min (y = 0.0031x^2^ + 0.0373x + 1.5641; R^2^ = 0.9733), 30 min (y = 0.0114x^2^ + 0.0235x + 1.5778; R^2^ = 0.9752).

**Figure 2:**
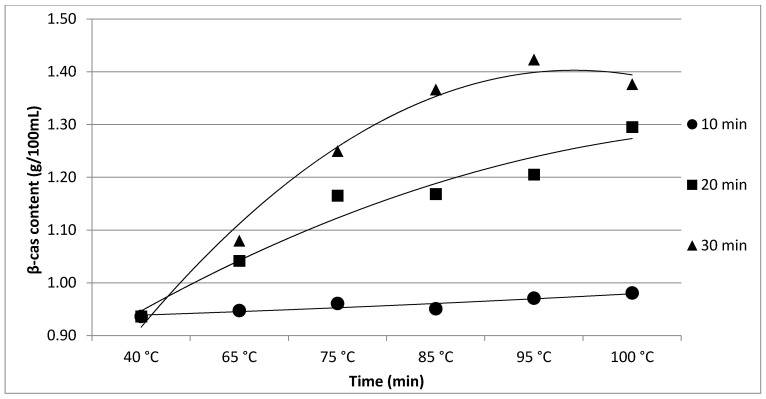
Effect of heating time and temperature on β-cas content. Equations: 10 min (y = 0.0004x^2^ + 0.0055x + 0.9329; R^2^ = 0.8644), 20 min (y = −0.0076x^2^ + 0.1184x + 0.8358; R^2^ = 0.9522), 30 min (y = −0.0251x^2^ + 0.2711x + 0.6698; R^2^ = 0.9861).

**Figure 3:**
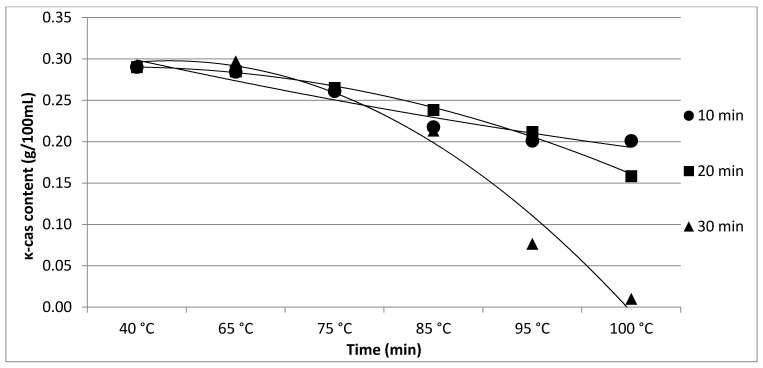
Effect of heating time and temperature on κ-cas content. Equations: 10 min (y = 0.001x^2^ − 0.0281x + 0.3257; R^2^ = 0.9307), 20 min (y = −0.0048x^2^ + 0.0077x + 0.2874; R^2^ = 0,9956), 30 min (y = −0.0139x^2^ + 0.0371x + 0.2731; R^2^ = 0.9768).

**Figure 4:**
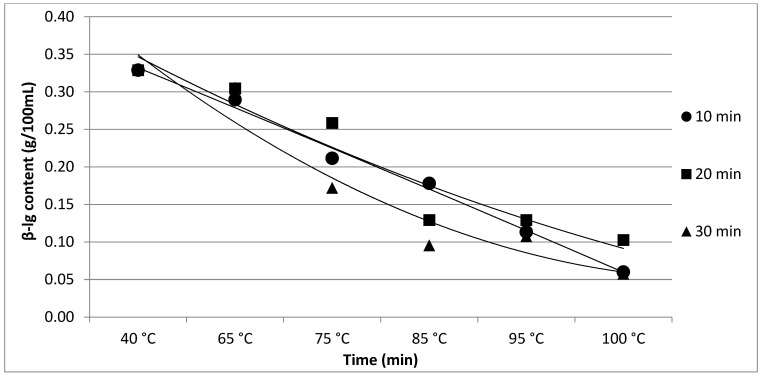
Effect of heating time and temperature on β-lg content. Equations: 10 min (y = −0.0003x^2^ – 0.0523x + 0.3845; R^2^ = 0.9929), 20 min (y = 0.0031x^2^ − 0.0725x + 0.416; R^2^ = 0.9191), 30 min (y = 0.008x^2^ −0.1141x + 0.4553; R^2^ = 0.935).

**Figure 5:**
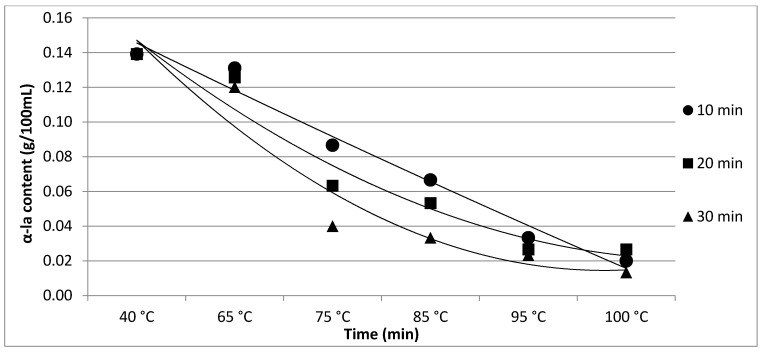
Effect of heating time and temperature on α-la content. Equations: 10 min (y = 0.0003x^2^ – 0.0283x + 0.1735; R^2^ = 0.9753), 20 min (y = 0.0038x^2^ − 0.0512x + 0.1946; R^2^ = 0.9497), 30 min (y = 0.0058x^2^ − 0.0672x + 0.2085; R^2^ = 0.9323).

The results of Experiment 1 are presented in Table 1. The effect of heating time and temperature on individual protein fractions’ content is visualized in Figure 1, Figure 2, Figure 3, Figure 4 and Figure 5. We found a significant (*p* < 0.05) effect of heating time and temperature on α-cas, β-cas, κ-cas, β-lg and α-la protein fractions.

The protein content in skimmed milk used for Experiment 1 and Experiment 2 was 3.40 ± 0.02 g/100 mL. The concentration of individual protein fraction in skimmed milk (Table 1) was: 1.60 ± 0.02 g/100 mL (α-cas), 0.94 ± 0.02 g/100 mL (β-cas), 0.29 ± 0.02 g/100 mL (κ-cas), 0.33 ± 0.02 g/100 mL (β-lg) and 0.14 ± 0.02 g/100 mL (α-la). After recalculation to the relative values %, the concentration of individual protein fraction in skimmed milk was: 46.82% (α-cas), 27.59% (β-cas), 21.17% (κ-cas), 9.84% (β-lg) and 4.23% (α-la). Similar results were reported by [2]: 15–19 g/L (α-cas), 9–11 g/L (β-cas), 2–4 g/L (κ-cas), 2–4 g/L (β-lg) and 0.6–1.7 g/L (α-la).

**Table 1:** Effect of heating time and temperature on the content of protein fractions (g/100 mL ± SD).

Heating Time	Protein Fraction	Heating Temperature						
		Raw	65 °C	75 °C	85 °C	95 °C	100 °C	*p*-Value
10 min	α-cas	1.60 ^c^	1.62 ^c^	1.63 ^c^	1.71 ^b^	1.75 ^ab^	1.80 ^a^	*p* < 0.05
		±0.02	±0.02	±0.03	±0.02	±0.01	±0.08	
	β-cas	0.94 ^c^	0.95 ^bc^	0.96 ^abc^	0.95 ^bc^	0.97 ^ab^	0.98 ^a^	*p* < 0.05
		±0.02	±0.03	±0.01	±0.03	±0.01	±0.02	
	κ-cas	0.29 ^a^	0.28 ^a^	0.26 ^ab^	0.22 ^bc^	0.20 ^c^	0.20 ^c^	*p* < 0.05
		±0.02	±0.03	±0.02	±0.03	±0.01	±0.02	
	β-lg	0.33 ^a^	0.29 ^ab^	0.21 ^bc^	0.18 ^cd^	0.11 ^de^	0.06 ^e^	*p* < 0.05
		±0.02	±0.03	±0.04	±0.04	±0.03	±0.05	
	α-la	0.14 ^a^	0.13 ^a^	0.09 ^b^	0.07 ^c^	0.03 ^d^	0.02 ^d^	*p* < 0.05
		±0.02	±0.03	±0.02	±0.04	±0.01	±0.05	
20 min	α-cas	1.60 ^d^	1.66 ^cd^	1.71 ^bcd^	1.78 ^bc^	1.79 ^ab^	1.91 ^a^	*p* < 0.05
		±0.04	±0.01	±0.01	±0.02	±0.02	±0.02	
	β-cas	0.94 ^d^	1.04 ^c^	1.17 ^b^	1.17 ^b^	1.21 ^b^	1.30 ^a^	*p* < 0.05
		±0.02	±0.03	±0.01	±0.04	±0.02	±0.03	
	κ-cas	0.29 ^a^	0.29 ^a^	0.27 ^ab^	0.24 ^bc^	0.21 ^c^	0.16 ^d^	*p* < 0.05
		±0.02	±0.02	±0.03	±0.02	±0.01	±0.02	
	β-lg	0.33 ^a^	0.30 ^ab^	0.26 ^abc^	0.13 ^bc^	0.13 ^bc^	0.10 ^c^	*p* < 0.05
		±0.03	±0.02	±0.03	±0.02	±0.05	±0.04	
	α-la	0.14^a^	0.13 ^a^	0.06 ^b^	0.05 ^b^	0.03 ^c^	0.03 ^c^	*p* < 0.05
		±0.02	±0.02	±0.02	±0.03	±0.02	±0.03	
30 min	α-cas	1.60 ^e^	1.69 ^d^	1.77 ^dc^	1.80 ^c^	2.01 ^b^	2.13 ^a^	*p* < 0.05
		±0.02	±0.03	±0.02	±0.03	±0.03	±0.02	
	β-cas	0.94 ^e^	1.08 ^d^	1.25 ^c^	1.37 ^b^	1.42 ^a^	1.38 ^b^	*p* < 0.05
		±0.03	±0.03	±0.04	±0.02	±0.04	±0.05	
	κ-cas	0.29 ^a^	0.30 ^a^	0.26 ^a^	0.21 ^b^	0.08 ^c^	0.01 ^d^	*p* < 0.05
		±0.03	±0.02	±0.03	±0.03	±0.02	±0.05	
	β-lg	0.33 ^a^	0.31 ^a^	0.17 ^b^	0.10 ^b^	0.11 ^b^	0.06 ^b^	*p* < 0.05
		±0.03	±0.02	±0.03	±0.06	±0.05	±0.04	
	α-la	0.14 ^a^	0.12 ^a^	0.04 ^b^	0.03 ^bc^	0.02 ^bc^	0.01 ^d^	*p* < 0.05
		±0.03	±0.03	±0.04	±0.03	±0.02	±0.04	

^a–e^ Results assigned a different superscript letter are statistically significantly different (Tukey’s test, *p* < 0.05).

**Figure 6:**
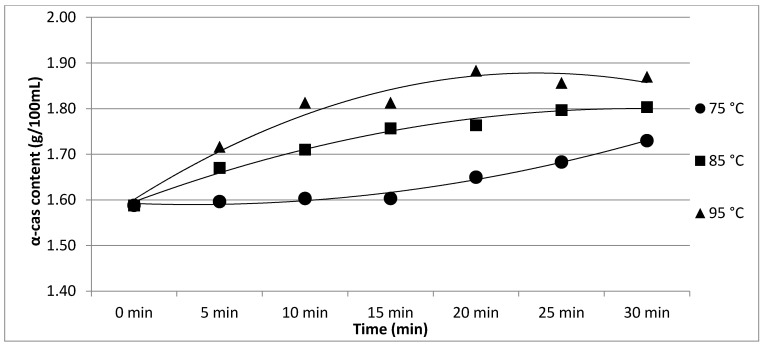
Effect of heating temperature and time on α-cas content. Equations: 75 °C (y = 0.005x^2^ − 0.0168x + 1.604; R^2^ = 0.9836), 85 °C (y = −0.0058x^2^ + 0.0809x + 1.5209; R^2^ = 0.9868), 95 °C (y = −0.0125x^2^ + 0.1429x + 1.4706; R^2^ = 0.9624).

**Figure 7:**
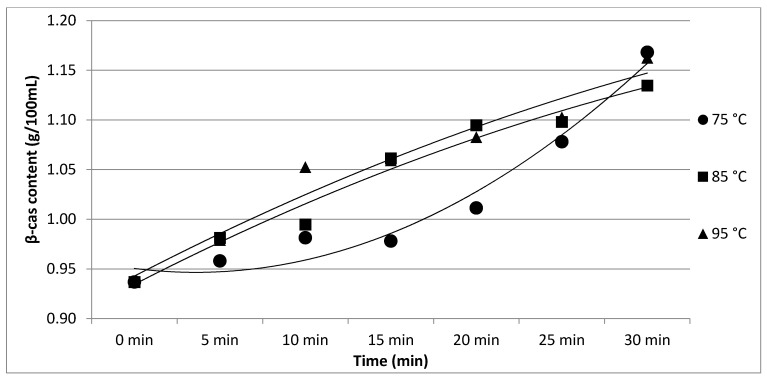
Effect of heating temperature and time on β-cas content. Equations: 75 °C (y = 0.0075x^2^ − 0.0259x + 0.9688; R^2^ = 0.9667), 85 °C (y = −0.0019x^2^ + 0.0479x + 0.8884; R^2^ = 0.9725), 95 °C (y = −0.0017x^2^ + 0.0479x + 0.8966; R^2^ = 0.9545).

**Figure 8:**
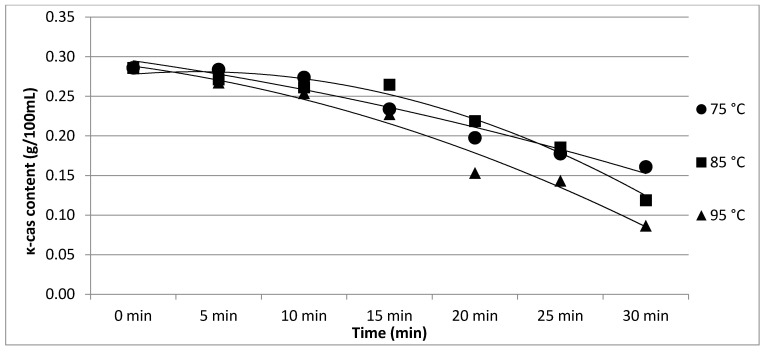
Effect of heating temperature and time on κ-cas content. Equations: 75 °C (y = −0.0014x^2^ − 0.0128x + 0.3089; R^2^ = 0.9616), 85 °C (y = −0.0057x^2^ + 0.0198x + 0.2638; R^2^ = 0.9764), 95 °C (y = −0.0032x^2^ − 0.0082x + 0.2995; R^2^ = 0.9727).

**Figure 9:**
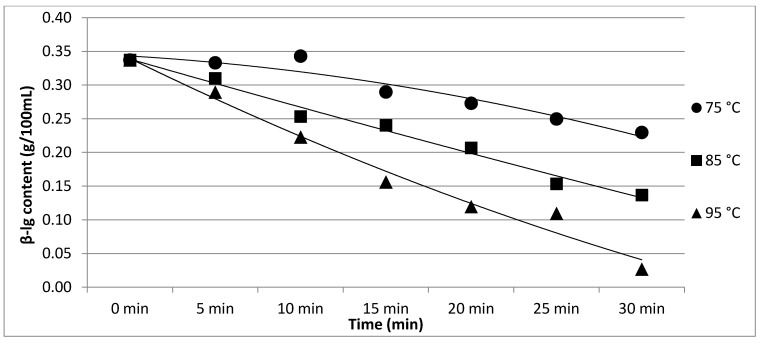
Effect of heating temperature and time on β-lg content. Equations: 75 °C (y = −0.0021x^2^ − 0.0035x + 0.3488; R^2^ = 0.933), 85 °C (y = 0.0003x^2^ − 0.037x + 0.3753; R^2^ = 0.9842), 95 °C (y = 0.002x^2^ − 0.0658x + 0.4034; R^2^ = 0.9802).

**Figure 10:**
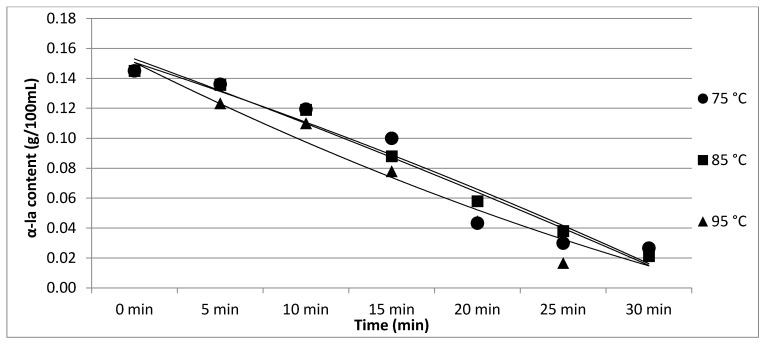
Effect of heating temperature and time on β-lg content. Equations: 75 °C (y = −0.0004x^2^ − 0.0202x + 0.1735; R^2^ = 0.9373), 85 °C (y = −0.0006x^2^ − 0.0175x + 0.1688; R^2^ = 0.9845), 95 °C (y = 0.001x^2^ − 0.0305x + 0.1802; R^2^ = 0.9566).

The results of Experiment 2 are presented in Table 2. The effect of heating temperature and time on individual protein fractions’ content is visualized in Figure 6, Figure 7, Figure 8, Figure 9 and Figure 10. We found a significant effect (*p* < 0.05) of heating temperature and time on α-cas, β-cas, κ-cas, β-lg and α-la protein fractions.

**Table 2:** Effect of heating temperature and time on the content of protein fractions (g/100mL ± SD).

Heating Temperature	Protein Fraction	Heating Time							
		Raw Milk	5 min	10 min	15 min	20 min	25 min	30 min	*p*-Value
75 °C	α-cas	1.59 ^b^	1.60 ^b^	1.60 ^b^	1.60 ^b^	1.65 ^ab^	1.68 ^ab^	1.73 ^a^	*p* < 0.05
		±0.07	±0.05	±0.02	±0.04	±0.02	±0.04	±0.07	
	β-cas	0.94 ^c^	0.96 ^c^	0.98 ^c^	0.98 ^c^	1.01 ^bc^	1.08 ^b^	1.17 ^a^	*p* < 0.05
		±0.04	±0.04	±0.03	±0.04	±0.03	±0.04	±0.10	
	κ-cas	0.29 ^a^	0.28 ^a^	0.27 ^a^	0.23 ^ab^	0.20 ^bc^	0.18 ^c^	0.16 ^c^	*p* < 0.05
		±0.04	±0.04	±0.04	±0.05	±0.05	±0.04	±0.04	
	β-lg	0.34 ^a^	0.33 ^a^	0.34 ^a^	0.29 ^b^	0.27 ^bc^	0.25 ^cd^	0.23 ^d^	*p* < 0.05
		±0.04	±0.03	±0.03	±0.04	±0.03	±0.04	±0.05	
	α-la	0.15 ^a^	0.14 ^a^	0.12 ^a^	0.10 ^ab^	0.04 ^bc^	0.03 ^c^	0.03 ^c^	*p* < 0.05
		±0.03	±0.03	±0.04	±0.03	±0.03	±0.03	±0.02	
85 °C	α-cas	1.59 ^e^	1.67 ^d^	1.71 ^c^	1.76 ^b^	1.76 ^b^	1.80 ^a^	1.80 ^a^	*p* < 0.05
		±0.04	±0.05	±0.06	±0.04	±0.03	±0.04	±0.03	
	β-cas	0.94 ^e^	0.98 ^d^	0.99 ^d^	1.06 ^c^	1.09 ^b^	1.10 ^b^	1.13 ^a^	*p* < 0.05
		±0.04	±0.04	±0.03	±0.04	±0.03	±0.05	±0.03	
	κ-cas	0.29 ^a^	0.27 ^a^	0.26 ^a^	0.26 ^a^	0.22 ^ab^	0.19 ^b^	0.12 ^c^	*p* < 0.05
		±0.04	±0.05	±0.03	±0.04	±0.03	±0.04	±0.05	
	β-lg	0.34 ^a^	0.31 ^b^	0.25 ^c^	0.24 ^c^	0.21 ^d^	0.15 ^e^	0.14 ^e^	*p* < 0.05
		±0.04	±0.04	±0.03	±0.04	±0.04	±0.03	±0.04	
	α-la	0.15 ^a^	0.14 ^a^	0.12 ^ab^	0.09 ^bc^	0.06 ^cd^	0.04 ^d^	0.02 ^d^	*p* < 0.05
		±0.03	±0.03	±0.04	±0.05	±0.04	±0.03	±0.03	
95 °C	α-cas	1.59 ^d^	1.72 ^c^	1.81 ^b^	1.81 ^b^	1.88 ^a^	1.86 ^a^	1.87 ^a^	*p* < 0.05
		±0.04	±0.05	±0.04	±0.03	±0.04	±0.05	±0.04	
	β-cas	0.94 ^f^	0.98 ^e^	1.05 ^d^	1.06 ^cd^	1.08 ^bc^	1.10 ^b^	1.16 ^a^	*p* < 0.05
		±0.03	±0.03	±0.04	±0.04	±0.04	±0.04	±0.03	
	κ-cas	0.29 ^a^	0.27 ^ab^	0.25 ^ab^	0.23 ^b^	0.15 ^c^	0.14 ^c^	0.09 ^d^	*p* = 0.10
		±0.05	±0.04	±0.06	±0.04	±0.05	±0.04	0.100	
	β-lg	0.34 ^a^	0.29 ^b^	0.22 ^c^	0.165 ^d^	0.12 ^e^	0.11 ^e^	0.03 ^f^	*p* < 0.05
		±0.04	±0.04	±0.03	±0.04	±0.04	±0.06	±0.05	
	α-la	0.15 ^a^	0.12 ^a^	0.11 ^a^	0.08 ^b^	0.04 ^c^	0.02 ^c^	0.03 ^c^	*p* < 0.05
		±0.03	±0.03	±0.05	±0.03	±0.05	±0.03	±0.05	

^a–f^ Results assigned a different superscript letter are statistically significantly different (Tukey’s test, *p* < 0.05).

**Table 3:** Results of two-way ANOVA.

Protein Fraction	Two-Way ANOVA (*p*-Value)		
	Factor Temperature	Factor Time	Factor Interaction (Temperature × Time)
Experiment 1			
α-cas	<0.05 (0.00)	<0.05 (0.00)	<0.05 (0.00)
β-cas	<0.05 (0.00)	<0.05 (0.00)	<0.05 (0.00)
κ-cas	<0.05 (0.00)	<0.05 (0.00)	<0.05 (0.00)
β-lg	<0.05 (0.00)	<0.05 (0.00)	<0.05 (0.00)
α-la	<0.05 (0.00)	<0.05 (0.00)	<0.05 (0.00)
Experiment 2			
α-cas	<0.05 (0.00)	<0.05 (0.00)	<0.05 (0.00)
β-cas	<0.05 (0.00)	<0.05 (0.00)	<0.05 (0.00)
κ-cas	<0.05 (0.00)	<0.05 (0.00)	<0.05 (0.00)
β-lg	<0.05 (0.00)	<0.05 (0.00)	<0.05 (0.00)
α-la	<0.05 (0.00)	<0.05 (0.00)	<0.05 (0.00)

The effect of heat-treatment time and temperature on the protein markers used in the laboratory methods for analyzing the adulteration of milk cheese, such as the isoelectric focusing of gamma caseins [4], must be taken into consideration. Protein denaturation and complex formation can be sources of measurement uncertainty in native PAGE, SDS-PAGE or isoelectric focusing used to analyze milk and cheese products.

To better interpret the results, we calculated the percentage (%) increase or decrease in each protein fraction depending on the heating temperature and time. The results of denaturation degree represent the percentage calculation (%) are presented in Table 4.

**Figure 11:**
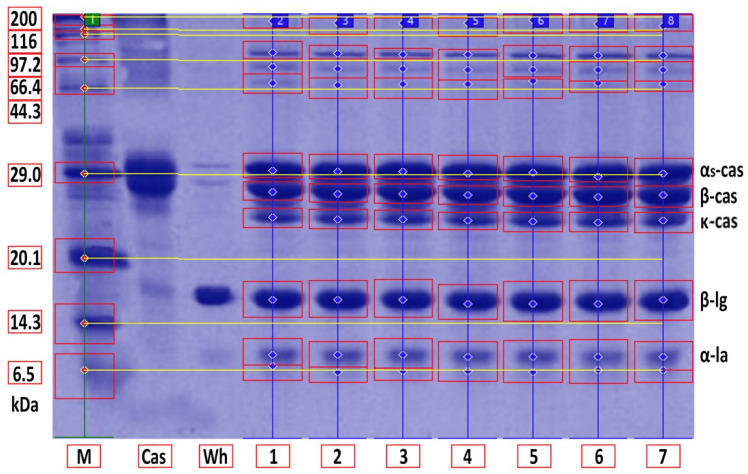
SDS-PAGE of whey and casein protein fractions after heating at 75 °C for different times. M—molecular marker; Cas—casein; Wh—whey protein; Lanes 1–7: raw milk, milk heat-treated for 5, 10, 15, 20, 25 and 30 min.

Whey protein in raw milk is characterized by poor thermal stability and can denature to different degrees [9]. The protein profile presented by the casein: whey ratio had a considerable impact on the physical characteristics of heat-treated milk [34]. Based on the data obtained from individual measurements taken during the heat treatment of milk samples with an increasing trend of temperature (65, 75, 85, 95 and 100 °C) for different times (10, 20 and 30 min), certain trends in milk protein concentration in terms of the proportions of α-cas, β-cas, κ-cas, β-lg and α-la were identified, in direct proportion with increasing temperature and heating time of the milk samples. For α-cas, with an initial concentration of 1.60 ± 0.02 g/100mL for raw milk, we recorded an increase in concentration as the length and temperature of heating increased, with the highest value of 2.13 ± 0.02 g/100 mL recorded at 100 °C and a heating time of 30 min. A substantially similar trend was observed for β-cas, with a value of 0.94 ± 0.02 g/100 mL in the untreated milk sample, and a maximum value of 1.38 ± 0.05 g/100 mL reached at 100 °C and 30 min heating time. For κ-cas, we recorded the opposite trend to that for α- and β-cas, with a value of 0.29 ± 0.02 µg for the untreated sample, and the lowest value of 0.01 ± 0.05 µg for the longest heating at the highest temperature. For β-lg (0.33 ± 0.02 µg) and α-la (0.14 ± 0.02 µg), the highest values were recorded for the untreated sample; gradual heating at specified lengths and temperatures decreased the concentration of both proteins. A similar trend as presented in Table 1 is shown in Table 2, taking into account two established factors: heating temperature (75, 85 and 95 °C) and duration of heat treatment (between 5 and 30 min, in 5 min increments). A more gradual increase in the duration of heat treatment uncovered the breakpoint for the concentration of proteins, which varied from protein to protein but showed the same trend as observed during the longest heat treatment. In this way, different preheating temperatures have been shown to alter the degree of milk whey protein denaturation and improve the heat stability of proteins [17,26,28,35]. The critical breakpoints for individual protein fractions were analyzed in our study. The degree of denaturation was determined by the relative amounts of native whey protein in unheated milk compared to those in samples after heat treatment ranging from 65 to 100 °C (every 10 °C rise in temperature during treatment for 10, 20 and 30 min), presented by SDS-PAGE. Figure 11 shows that the whey protein bands of milk changed from intense to faint under treatment at different temperatures (equal to protein concentration), which means whey protein denatured differently at different temperatures. These findings follow those of other authors [9]. Compared with untreated raw milk, the total whey protein content was decreased in the supernatant fluid by heat treatment. At the same temperature, whey protein bands in milk were most intense when heat treatment lasted 10 min and faintest after heat treatment for 30 min (prolonged heat treatment causes a greater degree of whey protein denaturation) [9]. The denaturation of whey proteins exists in two forms: soluble protein aggregates and aggregation on the surface of the casein micelle [36].

The following denaturation degree of individual proteins fractions (Table 4) was found at pasteurization temperatures: 75 °C during 5 min: α-cas (+0.5%), β-cas (+2.3%), κ-cas (−0.8%), β-lg (−1.2%), α-la (−6.2%); 85 °C during 5 min: α-cas (+5.2%), β-cas (+4.7%), κ-cas (−5.1%), β-lg (−8%), α-la (−6.5%); 95 °C during 5 min: α-cas (+8.1%), β-cas (+4.5%), κ-cas (−6.5%), β-lg (−14.2%), α-la (−14.9%). On the basis of these results, we can state the increase in denaturation degree in whole protein fractions under the increase in pasteurization temperature; the same effect was recorded for pasteurization time increase as well.

Denaturation of heat-labile whey proteins is known to be initiated on increasing the temperature to >~62 (α-la) and ~75 °C (β-lg) [37]. In addition, the α-la bands almost disappeared for 6 kDa (Figure 11), especially at 95 °C; this was caused by the formation of an α-la and casein complex [38] and can be explained by the poor thermal stability of whey protein during the heat treatment of milk [39]. Also, the effect of evaporation during the heat treatment should be taken into account. The casein–lactoglobulin complex was detected as casein by the analytical technique SDS page used at 29 kDa (Figure 11). The increase in α-cas band density was probably due to the decrease in the mass of α-la, β-lg due to the thermal denaturation of these whey α-la and β-lg fractions. Specific treatment in the samples to dissolve these complexes was applied (SDS sample buffer-reducing solution). Moreover, the complexation increased the size and therefore changed the zone profiles on the gel. Increase in concentration can be caused by evaporation during heat treatment.

With an increase in treatment temperature, the degree to which whey protein combines with casein increases—a mechanism approved by [40]. However, the bands of β-lg were relatively intense at 75 °C (low degree of combination of whey protein with casein) [9]; similar research confirmed that heat treatment at a higher temperature and for a long time can make the hydrophobic groups in the protein interact, thus promoting the combination of β-lg with κ-cas [41]. Based on the results of our study, the detected degree of combination of whey protein with casein was almost the same under the heat treatment of milk samples for 5 min at 75, 85 and 95 °C [9]. In general, the interpretation of the intensity level of milk protein bands revealed the ability of whey proteins (α-la and β-lg) to combine with casein and generate a stable polymer by disulphide bonds, leading to a change in the size of casein micelles [13], although casein is relatively stable [9]. The mechanism by which milk whey protein changes during heating is also described by [42], who pointed to changes through the thiol and disulphide bonds in intermolecular disulphide bond exchange [9]. Furthermore, the disulphide bonds, which are formed between casein and whey protein or whey protein with itself when milk is heat-treated, play a key role in aggregating milk compounds during heat treatment [43].

In conclusion, heat treatment at temperatures above 60 °C leads to whey protein denaturation, coupled with the formation of whey protein polymer by itself or combined with casein. Almost all whey protein was denatured when milk was heated at 95 °C for 10 min in [9]. We agree with these results, and we found the same effect. From a practical point of view, it is necessary to take into consideration the effect of heat treatment on individual protein fractions which can be used as markers for the analysis of the adulteration of cheese products.

## 4. Conclusions

The content of the individual protein fractions in skimmed milk were: 1.60 ± 0.02 g/100 mL (α-cas), 0.94 ± 0.02 g/100 mL (β-cas), 0.29 ± 0.02 g/100 mL (κ-cas), 0.33 ± 0.02 g/100 mL (β-lg) and 0.14 ± 0.02 g/100 mL (α-la). The whey protein denaturation process and the formation of the whey protein–casein complex depended significantly (*p* < 0.05) on an increase in heat-treatment time and temperature. The composition of all individual protein fractions were changed. Generally, the κ-cas, β-lg and α-la protein fractions decreased. The following denaturation degrees of individual proteins fractions were found at a pasteurization temperature of 85 °C at 5 min: α-cas (+5.2%), β-cas (+4.7%), κ-cas (−5.1%), β-lg (−8%) and α-la (−6.5%). Prolonging the heat-treatment time in conjunction with an increase in heat-treatment temperature increases the degree to which whey protein is denatured and forms a whey protein–casein complex. This finding can be utilized by the dairy industry to set up appropriate heat-treatment conditions to secure the stability of the milk protein and preserve its nutritional value. Another important finding is that there was a change in the content of β-cas, which is used after the cleavage to the gamma 2 and 3 caseins markers for the detection of adulteration in some dairy products.

## Figures and Tables

**Table 4:** Protein denaturation degree (%).

**Experiment 1**							
**Time**	**Protein**	**Temperature**					
		**65 °C**	**75 °C**	**85 °C**	**95 °C**	**100 °C**	**-**
10 min	α-cas	1.2	2.3	7.1	9.8	12.7	-
	β-cas	1.2	2.6	1.6	3.7	4.8	-
	κ-cas	−2.0	−10.0	−25.0	−30.7	−30.7	-
	β-lg	−12.0	−35.7	−45.8	−65.5	−81.8	-
	α-la	−5.8	−37.8	−52.1	−76.1	−85.6	-
20 min	α-cas	3.7	6.8	11.2	12.2	19.7	-
	β-cas	11.3	24.4	24.8	28.7	38.3	-
	κ-cas	−1.7	−8.6	−17.8	−27.0	−45.4	-
	β-lg	−7.4	−21.4	−60.7	−60.7	−68.8	-
	α-la	−9.7	−54.5	−61.7	−80.8	−80.8	-
30 min	α-cas	5.9	10.9	12.6	25.5	33.0	-
	β-cas	15.3	33.5	45.9	52.0	47.0	-
	κ-cas	2.4	−9.3	−26.5	−73.6	−96.6	-
	β-lg	−7.1	−47.7	−71.0	−67.3	−82.5	-
	α-la	−13.7	−71.3	−76.1	−83.2	−90.4	-
**Experiment 2**							
**Temperature**	**Protein**	**Time**					
		**5 min**	**10 min**	**15 min**	**20 min**	**25 min**	**30 min**
75 °C	α-cas	0.5	0.4	0.0	2.9	2.0	2.8
	β-cas	2.3	2.4	−0.3	3.4	6.6	8.3
	κ-cas	−0.8	−3.5	−14.6	−15.5	−10.1	−9.4
	β-lg	−1.2	3.0	−15.6	−5.8	−8.5	−8.0
	α-la	−6.2	−12.2	−16.3	−56.7	−30.8	−11.1
85 °C	α-cas	5.2	2.4	2.7	0.4	1.9	0.4
	β-cas	4.7	1.4	6.7	3.1	0.3	3.3
	κ-cas	−5.1	−3.7	1.3	−17.4	−15.2	−35.9
	β-lg	−8.0	−18.3	−5.3	−13.9	−25.8	−10.9
	α-la	−6.5	−12.3	−26.0	−34.1	−34.5	−43.8
95 °C	α-cas	8.1	5.6	0.0	3.9	−1.4	0.7
	β-cas	4.5	7.5	0.6	2.2	1.8	5.4
	κ-cas	−6.5	−5.0	−10.5	−32.6	−6.5	−39.5
	β-lg	−14.2	−23.0	−29.9	−23.5	−8.4	−75.6
	α-la	−14.9	−10.8	−29.2	−42.8	−62.6	−60.0

Note: the protein denaturation degree represents increase or decrease in the protein fraction depending on the heating temperature and time.

## Data Availability

Data is contained within the article.
